# Chronic respiratory disease in adult outpatients in three African countries: a cross-sectional study

**DOI:** 10.5588/ijtld.21.0362

**Published:** 2022-01-01

**Authors:** A. B. Binegdie, H. Meme, A. El Sony, T. Haile, R. Osman, B. Miheso, L. Zurba, M. Lesosky, J. Balmes, P. J. Burney, K. Mortimer, G. Devereux

**Affiliations:** 1 Division of Pulmonary and Critical Care Medicine, Department of Internal Medicine, College of Health Sciences, Addis Ababa University, Addis Ababa, Ethiopia; 2 Centre for Respiratory Diseases Research, Kenyan Medical Research Institute (KEMRI), Nairobi, Kenya; 3 Epidemiological Laboratory (Epi-Lab) for Public Health, Research and Development, Khartoum, Sudan; 4 Education for Health Africa, Durban, South Africa; 5 Division of Epidemiology Biostatistics, School of Public Health & Family Medicine, University of Cape Town, Cape Town, South Africa; 6 University of California, San Francisco, CA, USA; 7 National Heart and Lung Institute, Imperial College London, London, UK; 8 Liverpool School of Tropical Medicine, Liverpool, UK

**Keywords:** chronic respiratory symptoms, spirometry, COPD, asthma, Africa, Ethiopia, Kenya, Sudan, hospital clinics

## Abstract

**BACKGROUND:**

The greatest burden of chronic respiratory disease is in low- and middle-income countries, with recent population-based studies reporting substantial levels of obstructive and restrictive lung function.

**OBJECTIVE:**

To characterise the common chronic respiratory diseases encountered in hospital outpatient clinics in three African countries.

**METHODS:**

This was a cross-sectional study of consecutive adult patients with chronic respiratory symptoms (>8 weeks) attending hospital outpatient departments in Ethiopia, Kenya and Sudan. Patients were assessed using a respiratory questionnaire, spirometry and chest radiography. The diagnoses of the reviewing clinicians were ascertained.

**RESULT:**

A total of 519 patients (209 Kenya, 170 Ethiopia, 140 Sudan) participated; the mean age was 45.2 years (SD 16.2); 53% were women, 83% had never smoked. Reviewing clinicians considered that 36% (95% CI 32–40) of patients had asthma, 25% (95% CI 21–29) had chronic bronchitis, 8% (95% CI 6–11) chronic obstructive pulmonary disease (COPD), 5% (95% CI 4–8) bronchiectasis and 4% (95% CI 3–6) post-TB lung disease. Spirometry consistent with COPD was present in 35% (95% CI 30–39). Restriction was evident in 38% (95% CI 33–43). There was evidence of sub-optimal diagnosis of asthma and COPD.

**CONCLUSION:**

In Ethiopia, Kenya and Sudan, asthma, COPD and chronic bronchitis account for the majority of diagnoses in non-TB patients with chronic respiratory symptoms. The suboptimal diagnosis of these conditions will require the widespread use of spirometry.

## Abstract (fr)

**CONTEXTE:**

Les pays à revenu faible et intermédiaire portent la charge la plus lourde en matière de maladies respiratoires chroniques. De récentes études populationnelles ont rapporté des taux considérables de maladies pulmonaires restrictives et obstructives.

**OBJECTIF:**

Caractériser les maladies respiratoires chroniques couramment rencontrées en milieu hospitalier ambulatoire dans trois pays africains.

**MÉTHODES:**

Il s’agissait d’une étude transversale réalisée auprès de patients adultes consécutifs présentant des symptômes respiratoires chroniques (>8 semaines) pris en charge dans des services hospitaliers ambulatoires en Éthiopie, au Kenya et au Soudan. Les patients ont été évalués par questionnaire respiratoire, spirométrie et radiographie thoracique. Les diagnostics des médecins-conseil ont été vérifiés.

**RÉSULATS:**

Au total, 519 patients (209 Kenya, 170 Éthiopie, 140 Soudan) ont participé à l’étude. L’âge moyen était de 45,2 ans (SD 16,2) ; 53% étaient des femmes et 83% n’avaient jamais fumé. Les médecinsconseil ont considéré que 36% (IC 95% 32–40) des patients avaient de l’asthme, 25% (IC 95% 21–29) une bronchite chronique, 8% (IC 95% 6–11) une bronchopneumopathie obstructive chronique (COPD), 5% (IC 95% 4–8) une bronchectasie et 4% (IC 95% 3–6) une maladie pulmonaire post-TB. Une spirométrie cohérente avec une COPD a été observée chez 35% (IC 95% 30–39) des patients. Une maladie restrictive était évidente chez 38% (IC 95% 33–43) des patients. Des données probantes de diagnostic sous-optimal d’asthme et de COPD ont été observées.

**CONCLUSION:**

En Éthiopie, au Kenya et au Soudan, l’asthme, la COPD et la bronchite chronique représentent la majorité des diagnostics établis chez les patients non atteints de TB présentant des symptômes respiratoires chroniques. Le diagnostic sous-optimal de ces pathologies nécessitera l’utilisation généralisée de la spirométrie.

Chronic respiratory diseases (CRDs) are leading noncommunicable diseases worldwide, most probably because of the ubiquity of poverty, and inhaled noxious environmental, occupational and behavioural exposures.^1^ CRDs affect more than 1 billion people worldwide, with asthma and chronic obstructive pulmonary disease (COPD) being the most prevalent: about 300 million people have asthma and 200 million have COPD.^1^–^3^ The greatest burden of CRDs occurs in low- and middle-income countries (LMICs): almost 90% of COPD deaths and 80% of asthma deaths occur in LMICs, where they have been linked with poverty, poor access to healthcare and limited health service resources.^4^ Capacity for CRD diagnosis and management, such as spirometry and inhaled therapies, are not generally available in primary health care in public health services.^5^–^8^

Among the 1.1 billion people who live in sub-Saharan Africa, population-based surveys report wide variations in asthma symptom prevalence (5.7–20.3%), being highest in ‘westernised’ urban settings.^9^ The Burden of Obstructive Lung Disease (BOLD) study reports that the prevalence of fixed airflow obstruction in sub-Saharan African countries ranges from 9–73% in men and 4–86% in women.^10^ BOLD and other studies also report variations in the prevalence of restricted spirometry (4–49%).^11^–^13^

The aim of this clinic-based study was to describe and estimate the prevalence of common CRDs in patients with chronic respiratory symptoms attending hospital outpatient departments in three sub-Saharan African countries.

## METHODS AND MATERIALS

### Study design and setting

Between June 2019 and March 2020, a cross-sectional study was conducted in the outpatient departments of general hospitals in three sub-Saharan African countries: Bishofitu Hospital, Addis Ababa, Ethiopia; Mbagathi Hospital, Nairobi, Kenya; Shabb Hospital, Khartoum, Sudan.

Ethiopia, Kenya and Sudan are East African Countries with populations of respectively 112 million, 53 million and 43 million. Ethiopia and Sudan are among the world’s least developed countries (annual per capita income respectively US$855 and US$441) and Kenya is a lower-middle-income country (US$1,816/year).^14^

Consecutive adult patients aged ≥18 years with chronic respiratory symptoms (>8 weeks)^15^ reviewed in the participating hospital outpatient departments were invited to take part. Exclusion criteria wee clinical suspicion of TB, positive GeneXpert sputum test result, pregnancy, acute respiratory infection or contraindications for spirometry.

### Data collection

An interviewer administered a respiratory questionnaire based on that used in BOLD with additional questions developed as part of the National Institute for Health Research International Multidisciplinary Programme to Address Lung Health and TB in Africa “IMPALA”. The questions covered demographics, symptoms, medical history, exposures to outdoor and indoor pollutants, tobacco smoking and occupation.^16^ The diagnosis made by the reviewing clinician was ascertained.

Pre- and post-bronchodilator (BD) spirometry was performed as detailed below. A postero-anterior chest radiograph was also performed and systematically read/recorded by a radiologist.^17^

### Spirometry

Spirometry was conducted in accordance with Pan African Thoracic Society (PATS), American Thoracic Society/European Respiratory Society recommendations by trained and PATS-certified technicians/nurses in Competence in Foundational Spirometry.^18^,^19^ The EasyOne Spirometer (NDD Medizintechnik, Zurich, Switzerland) was used with daily calibration checks. Up to eight forced exhalation manoeuvres were performed while sitting. All traces were reviewed by an external assessor, and measurements graded A–C for acceptability and repeatability were selected for analysis. The spirometric parameters recorded were forced expiratory volume in 1 sec (FEV_1_), forced vital capacity (FVC) and the ratio (FEV_1_/FVC). Spirometry measurements were compared with 2012 Global Lung Initiative reference equations for ‘Black’ ethnicity.^20^ Patients’ lung function was categorised as follows: normal pre-BD FEV_1_/FVC ≥ LLN (lower limit of normal), FEV_1_ ≥ LLN and FVC ≥ LLN; fixed-airflow obstruction (COPD) post-BD FEV_1_/FVC < LLN; and restrictive pattern FVC < LLN. Significant BD reversibility was defined as an improvement in FEV_1_ ≥ 12% of baseline and ≥200 ml.^21^ Two patterns of spirometry were considered consistent with asthma: 1) significant reversibility with pre-BD airflow obstruction, FEV_1_/FVC < LLN (strict classification), 2) significant reversibility alone (looser classification).^22^ As per GOLD (Global Initiative for Chronic Obstructive Lung Disease) guidelines, post-BD airflow obstruction (FEV_1_/FVC < 0.7) was categorised into mild (FEV_1_ ≥80% predicted), moderate (80% > FEV_1_ ≥ 50% predicted), severe (50% > FEV_1_ ≥ 30% predicted) and very severe (FEV_1_ < 30% predicted).^21^

### Statistical considerations

Descriptive data are presented as percentages or mean, with standard deviation (SD) and 95% confidence intervals (CIs), where appropriate. Two operational definitions of asthma prevalence are presented ‘clinician diagnosed’ and ‘current asthma’ (wheeze in the last year).^22^,^23^ Between-group comparisons were performed using χ^2^, Fisher’s exact test, *t*-tests or analysis of variance, as appropriate; *P* < 0.05 was considered statistically significant. We aimed to recruit 200 participants from each country (total sample size of 600), this would enable the study to estimate disease prevalence with 2% precision assuming disease prevalence was 5–10%. Analyses were performed using IBM SPSS Statistics for Windows, v25.0 (IBM, Armonk, NY, USA).

### Ethics considerations

Ethics approvals were obtained from the relevant Ethics Committees: Addis Ababa University Institutional Review Board, Addis Ababa, Ethiopia (reference 062/18/IM); Kenya Medical Research Institute Scientific Ethics Review Unit, Nairobi, Kenya (reference 3761); National Research Ethics Review Committee, Khartoum, Sudan (reference 1–9–18); Liverpool School of Tropical Medicine Research Ethics Committee, Liverpool, UK: (reference: 18–048). Written informed consent was obtained from each participant before enrolment.

## RESULTS

In total, 519 patients (209 Kenya, 170 Ethiopia, 140 Sudan) participated; the numbers completing the different study assessments are presented in Supplementary Table S1. All completed the questionnaire; acceptable pre- and post-BD spirometry was available for respectively 448 and 447 patients. Overall and individual country participant demographic characteristics are presented in Table 1. Participants tended to be female (53%) and middle-aged (mean age: 45 years, SD 16), with a minority having ever smoked (17%). About 15% of patients lived in rural environments, and nearly a third (29%) reported that they had gone short of food in the previous year. HIV seropositivity was reported by 4.1% and a previous history of TB by 18%.

**Table 1:** Background demographics

	All (*n* = 519) *n* (%)	Kenya (*n* = 209) *n* (%)	Ethiopia (*n* = 170) *n* (%)	Sudan (*n* = 140) *n* (%)	*P* value
Female sex	275 (53)	119 (56.9)	102 (60.0)	54 (38.6)	<0.001
Age, years, mean ± SD	45.2 ± 16.2	41.2 ± 14.5	48.6 ± 16.0	47.3 ± 17.5	<0.001
Employment					
Regular paid employment	239 (46.1)	76 (36.4)	82 (48.2)	81 (57.9)	<0.001
Irregular paid employment	280 (53.9)	97 (46.4)	55 (32.5)	19 (13.6)	<0.001
Housewife	92 (17.7)	14 (6.8)	51 (30.0)	27 (20.6)	
Sales	66 (12.7)	50 (24.4)	4 (2.4)	12 (9.2)	
Labourer	57 (11.0)	36 (17.6)	12 (7.1)	9 (6.9)	
Professional	37 (7.1)	22 (10.7)	0	15 (11.5)	
Farming	24 (4.6)	12 (5.9)	5 (2.9)	7 (5.3)	
Insufficient food in last year	150 (28.9)	31 (14.8)	97 (57.1)	22 (15.7)	<0.001
Smoking status					
Never	431 (83.0)	178 (88.6)	157 (92.4)	96 (68.6)	<0.001
Tobacco smoke (home)	96 (18.5)	32 (15.3)	26 (15.3)	38 (27.1)	0.014
Tobacco smoke (work)	167 (32.2)	93 (44.5)	30 (17.6)	44 (31.4)	<0.001
Rural residence	73 (14.1)	41 (19.6)	15 (8.8)	17 (12.1)	0.008
BMI, kg/m^2^, mean ± SD	24.2 ± 5.61	24.4 ± 5.62	23.2 ± 4.85	25.0 ± 6.31	0.016
Underweight	88 (17.3)	32 (16.0)	27 (15.9)	29 (20.7)	0.006
Optimal weight	239 (46.9)	84 (42.0)	92 (54.1)	63 (45.0)	
Overweight	96 (18.8)	47 (23.5)	33 (19.4)	16 (11.4)	
Obese	87 (17.1)	37 (18.5)	18 (10.6)	32 (22.9)	
Past history of:					
TB	93 (17.9)	43 (20.6)	24 (14.1)	26 (18.6)	0.258
HIV-positive	21 (4.1)	20 (10)	1 (0.6)	0	<0.001
Patient reported doctor diagnosed					
Asthma	175 (33.9)	59 (28.2)	61 (35.9)	55 (40.1)	0.058
COPD*	23 (4.4)	1 (0.5)	6 (3.5)	16 (12.4)	<0.001
Chronic bronchitis	72 (14.1)	6 (2.9)	44 (25.9)	22 (16.9)	<0.001
Reviewing clinician diagnosis					
Asthma	186 (35.8)	65 (31.1)	66 (38.8)	55 (39.3)	0.181
COPD*	41 (7.9)	3 (1.4)	26 (15.3)	12 (8.6)	<0.001
Chronic bronchitis	129 (24.9)	47 (22.5)	72 (42.4)	10 (7.1)	<0.001

* 12 patients with diagnosed asthma also diagnosed with COPD.

SD = standard deviation; BMI = body mass index; COPD = chronic obstructive pulmonary disease.

The prevalence of respiratory symptoms is presented in Table 2. The prevalence of current asthma (wheeze in the last year) was 71% (95% CI 66–74), being highest in Ethiopia (92%, 95% CI 87–95) and lowest in Sudan (56%, 95% CI 48–65). Reviewing clinicians considered the main diagnosis to be asthma in 36% (95% CI 32–40), COPD in 8% (95% CI 6–11) and chronic bronchitis in 25% (95% CI 21–29). Other diagnoses included bronchiectasis (5%, 95% CI 4–8), fibrosis (5%, 95% CI 3–7) and post-TB lung disease (4%, 95% CI 3–6).

**Table 2:** Respiratory symptoms

	All (*n* = 519) n (%)	Kenya (*n* = 209) *n* (%)	Ethiopia (*n* = 170) *n* (%)	Sudan (*n* = 140) *n* (%)	*P* value
Symptoms					
Cough in the absence of a cold	345 (66.5)	94 (44.9)	138 (81.2)	113 (80.7)	<0.001
Usually expectorate sputum	269 (56.0)	93 (44.5)	115 (67.6)	61 (60.4)	<0.001
Wheeze in the last 12 months	366 (70.5)	131 (62.7)	156 (91.8)	79 (56.4)	<0.001
Short of breath, unable to dress/leave house	208 (40.5)	56 (26.8)	119 (70.0)	33 (24.4)	<0.001

### Lung function

In total, 426 (82%) patients provided acceptable and repeatable pre- and post-BD spirometry, and with the exception of an excess of cough and dyspnoea symptoms, these were similar to those unable/unwilling to undergo spirometry (Supplementary Table S2).

The mean FEV_1_ was 74% predicted (95% CI 72–77) and FVC 82% predicted (80–84%) (Table 3). About a third of patients had spirometry consistent with COPD: 35% (95% CI 30–39) had post-BD FEV_1_/FVC < LLN and 32% (95% CI 28–37) had post-BD FEV_1_/FVC < 0.70. Of the obstructed patients, 60% had no/insignificant BD reversibility and about a half had severe or very severe obstruction. Spirometric evidence of restriction was evident in 38% (95% CI 34–43). Overall classification was as follows: 33% (95% CI 29–38) had normal spirometry, 18% (95% CI 15–22) had pure obstruction, 17% (95% CI 14–21) had pure restriction, 23% (95% CI 19–28) had a mixed pattern (obstruction and restriction) and 8.0% (95% CI 5.6–11) were unclassifiable.

**Table 3:** Lung function of patients attending with respiratory symptoms (GLI 2012 reference)

	All *n* (%)	Kenya *n* (%)	Ethiopia *n* (%)	Sudan *n* (%)	*P* value
FEV_1_ pre-BD, %pred, mean % (95% CI)*	68.2 (65.7–70.7)	80.1 (76.5–83.7)	50.9 (47.2–54.6)	72.8 (68.1–77.5)	<0.001
FEV_1_ pre-BD <LLN	256 (57.1)	73 (41.2)	124 (81.0)	59 (50.0)	<0.001
FEV_1_ post-BD, %pred, mean % (95% CI)^†^	74.1 (71.7–76.6)	86.5 (83.1–90.0)	58.8 (55.1–62.4)	75.8 (71.3–80.4)	<0.001
FEV_1_ post-BD <LLN	209 (46.8)	48 (27.6)	111 (72.5)	50 (41.7)	<0.001
FVC post-BD, %pred, mean % (95% CI)	81.6 (79.5–83.8)	92.7 (89.9–95.5)	69.2 (65.8–72.5)	81.5 (77.5–85.5)	<0.001
Fixed airflow obstruction (FEV_1_/FVC < LLN)	154 (34.5)	40 (23.0)	78 (51.0)	36 (30.0)	<0.001
Fixed airflow obstruction + no reversibility	93 (13.7)	24 (13.8)	40 (26.2)	29 (24.2)	<0.001
Restriction FVC post-BD <LLN	170 (38.0)	27 (15.5)	97 (63.4)	46 (38.3)	<0.001
Fixed airflow obstruction (FEV_1_/FVC<70%)	143 (32.0)	35 (20.1)	74 (48.4)	34 (28.3	<0.001
Mild FEV_1_ (≥80%)	18 (12.6)	9 (25.7)	3 (4.2)	6 (17.6)	0.002
Moderate FEV_1_ (50–80%)	52 (36.4)	15 (42.9)	21 (29.6)	16 (47.1)	
Severe FEV_1_ (30–50%)	48 (33.6)	9 (25.7)	32 (45.1)	7 (20.6)	
Very severe FEV_1_ (<30%)	22 (15.4)	2 (5.7)	15 (21.1)	5 (14.7)	
Significant reversibility	109 (24.4)	41 (23.6)	54 (35.3)	14 (11.7)	<0.001
Classification of individuals^‡^					
Normal	142 (33.3)	75 (47.5)	23 (15.0)	44 (38.3)	<0.001
Pure obstruction	78 (18.3)	35 (22.2)	29 (19.0)	14 (12.2)	
Pure restriction	73 (17.1)	14 (8.9)	36 (23.5)	23 (20.0)	
Obstruction and restriction	99 (23.2)	13 (8.2)	61 (39.9)	25(21.7)	
Not normal, unclassifiable	34 (8.0)	21 (13.3)	4 (2.6)	9 (7.8)	

^*^ Acceptable pre-BD spirometry (*n* = 448): Kenya (*n* = 177), Ethiopia (*n* = 153), Sudan (*n* = 120).

^†^Acceptable post-BD spirometry (*n* = 447): Kenya (*n* = 174), Ethiopia (*n* = 153), Sudan (*n* = 120).

^‡^Using those with acceptable pre- and post-BD spirometry (*n* = 426): Kenya (*n* = 158), Ethiopia (*n* = 153), Sudan (*n* = 115).

GLI = Global Lung Initiative; FEV_1_ = forced expiratory volume in 1 sec; BD = bronchodilator; CI = confidence interval; LLN = lower limit of normal; FVC = forced vital capacity.

There appeared to be between-country differences in the patterns of lung function deficit, with lung function being lowest in Ethiopia and highest in Kenya. In Ethiopia, 51% (95% CI 43–59) fulfilled the spirometric definition of COPD, 51% of these had no/insignificant BD reversibility and 66% had severe or very severe obstruction, restriction was evident in 63% (95% CI 55–71). In contrast, in Kenya 23% (95% CI 17–29) fulfilled the spirometric definition of COPD, 60% of these had no/insignificant BD reversibility and 31% had severe or very severe obstruction, while restriction was evident in 15% (95% CI 10–21). Individual lung function patterns indicated that 15% (95% CI 9.8–22) of patients had normal spirometry in Ethiopia, 38% (95% CI 29–48) in Sudan and 48% (95% CI 29–56) in Kenya. The most common lung function deficit in Kenya was pure obstruction (22%, 95% CI 16–29), whereas a mixed obstructive/restrictive pattern was most common in Ethiopia (40%, 95% CI 32–48) and Sudan (22%, 95% CI 15–30).

Cough, sputum expectoration and breathlessness were associated with reduced FEV_1_, FVC, and evidence of obstruction and restriction (Supplementary Table S3). Breathlessness was associated with significant reversibility. Pure restriction was not associated with an excess of symptoms. Clinician-diagnosed asthma was associated with increased wheeze but not cough, sputum expectoration or breathlessness (Table 4). Overall, clinician-diagnosed asthma was not associated with any differences in FEV_1_, FVC, or FEV_1_/FVC, but it was associated with BD reversibility and pure obstructive pattern of spirometry (25% vs. 14%). ‘Current asthma’ (i.e., wheeze in last year) was associated with reduced FEV_1_, FVC, FEV_1_/FVC, BD reversibility and pure obstructive and mixed patterns of spirometry (Supplementary Table S3). Clinician-diagnosed COPD was associated with an increase in the symptoms of sputum expectoration and breathlessness but not cough or wheeze (Table 4). Clinician-diagnosed COPD was not associated with any significant differences in FEV_1_, FVC, FEV_1_/FVC; however, the number of people with diagnosed COPD was small (*n* = 41, 7.9%).

**Table 4:** Symptoms and lung function in patients with diagnosed asthma and COPD

	Clinician diagnosis of asthma			Diagnosed COPD		
	
	Yes (*n* = 186) *n* (%)	No (*n* = 333) *n* (%)	*P* value	Yes (*n* = 41) *n* (%)	No (*n* = 478) *n* (%)	*P* value
Cough in absence of cold	124 (66.7)	221 (66.4)	0.945	30 (73.2)	315 (65.9)	0.344
Productive cough	100 (56.8)	169 (55.6)	0.794	29 (78.4)	240 (54.2)	0.004
Wheeze in last 12 months	165 (88.7)	201 (60.4)	<0.001	30 (73.2)	336 (70.3)	0.698
Too breathless to leave house	82 (44.3)	126 (38.3)	0.182	25 (61.0)	183 (38.7)	0.005
Post-bronchodilator						
Mean FEV_1_ %pred, % (95% CI)	74.0 (70.3–77.7)	74.8 (71.7–77.8)	0.746	67.0 (59.0–75.0)	75.2 (72.7–77.6)	0.060
Mean FEV_1_/FVC, % (95% CI)	72.3 (70.0–75.8)	73.3 (73.4–76.3)	0.055	73.9 (70.4–77.3)	73.9 (72.6–75.2)	0.980
FEV_1_ < LLN	84 (45.2)	146 (44.9)	0.958	24 (58.5)	206 (43.8)	0.069
FEV_1_/FVC < LLN	64 (34.4)	100 (30.8)	0.396	14 (34.1)	150 (31.9)	0.769
Significant reversibility	57 (30.6)	65 (20.0)	0.007	8 (19.5)	114 (24.3)	0.494
Classification of lung function						
Normal	46 (28.9)	96 (36.0)	0.047	9 (25.0)	133 (34.1)	0.171
Obstructed	40 (25.2)	38 (14.2)		6 (16.7)	72 (18.5)	
Restricted	22 (13.8)	51 (19.1)		11 (30.6)	62 (15.9)	
Mixed	38 (23.9)	61 (22.8)		9 (25.0)	90 (23.1)	
Not classifiable	13 (8.2)	21 (7.9)		1 (2.8)	33 (8.5)	
Chest radiograph						
Normal	86 (59.3)	148 (56.5)	0.581	19 (61.3)	215 (57.2)	0.656
Consolidation	9 (6.2)	25 (9.5)	0.244	1 (3.2)	33 (8.8)	0.497
Hyperinflation	25 (17.2)	35 (13.4)	0.290	3 (9.7)	57 (15.2)	0.598
Fibrosis	5 (3.4)	28 (10.7)	0.010	4 (12.9)	29 (7.7)	0.309

COPD =chronic obstructive pulmonary disease; FEV_1_ = forced expiratory volume in 1 sec; CI = confidence interval; FVC = forced vital capacity; LLN = lower limit of normal.

### Lung function and diagnosis of airways disease

Given the lack of associations between clinician diagnoses of asthma or COPD and FEV_1_, and obstruction, further analyses were undertaken (Table 5). Of the 159 patients with clinician-diagnosed asthma and satisfactory spirometry, 62 (39%) fulfilled the spirometric criteria for COPD. Of the 267 patients without clinician-diagnosed asthma and satisfactory spirometry, about 20% had spirometry consistent with asthma: 47 (18%) had evidence of airflow obstruction and significant reversibility and 55 (21%) had significant reversibility alone (looser classification). Of the 36 patients with a previous COPD diagnosis and satisfactory spirometry, 23 (64%) did not fulfil the spirometric criteria for COPD (Table 6). Of the 390 without clinician-diagnosed COPD who provided satisfactory spirometry, 139 (36%) fulfilled the spirometric criteria for COPD, which is considerably more than those with clinician-diagnosed COPD.

**Table 5:** Patterns of airflow obstruction and reversibility in those with and those without clinician-diagnosed asthma

	All *n* (%)	Kenya *n* (%)	Ethiopia *n* (%)	Sudan *n* (%)
Clinician diagnosis of asthma, *n*	186	65	66	55
Asthma + acceptable spirometry, *n*	159	50	62	47
Asthma + reversibility*	52 (33)	21 (42)	23 (37)	8 (17)
Asthma + reversibility + AFO*	43 (27)	13 (26)	23 (37)	7 (15)
Asthma + no-reversibility + AFO	55 (35)	11 (22)	26 (42)	18 (38)
No diagnosis of asthma, *n*	333	144	104	85
No asthma + acceptable spirometry, *n*	267	108	91	68
No asthma + reversibility*	55 (21)	18 (17)	31 (34)	6 (9)
No asthma + reversibility + AFO*	47 (18)	11 (9)	31 (37)	5 (5)
No asthma + no reversibility + AFO	104 (39)	33 (31)	44 (48)	27 (40)

* ‘Asthma + reversibility + AFO’ is a sub-group of ‘Asthma + reversibility’.

AFO = airflow obstruction, i.e., post-bronchodilator FEV_1_/FVC<LLN; FEV_1_ = forced expiratory volume in 1 sec; FVC = forced vital capacity; LLN = lower limit of normal.

**Table 6:** Patterns of airflow obstruction in those with and those without clinician-diagnosed COPD

	All *n*	Kenya *n*	Ethiopia *n*	Sudan *n*
Clinicians’ diagnosis of COPD	41	3	26	12
COPD + acceptable spirometry	36	3	23	10
COPD + fixed AFO, *n* (%)				
COPD + no fixed AFO, *n* (%)	13 (36)	1 (33)	11 (48)	1 (10)
No diagnosis of COPD	23 (64)	2 (67)	12 (52)	9 (90)
COPD not diagnosed	478	206	144	128
No COPD +acceptable spirometry	390	155	130	105
No COPD + fixed AFO, *n* (%)	139 (36)	37 (24)	67 (52)	35 (33)

COPD = chronic obstructive pulmonary disease; AFO = airflow obstruction, i.e., post-bronchodilator FEV_1_/FVC<LLN; FEV_1_ = forced expiratory volume in 1 sec; FVC = forced vital capacity; LLN = lower limit of normal.

## DISCUSSION

This cross-sectional study was conducted in general hospitals of three African countries to estimate the prevalence of common CRDs in patients presenting to outpatient clinics with chronic respiratory symptoms. Consistent with the global CRD burden, the most common diseases reported by patients enrolled in this study were asthma, COPD and chronic bronchitis, accounting for about 70% of patients.^24^ The prevalence of clinician-diagnosed asthma and ‘current asthma’ (wheeze in the last year) were respectively 36% (95% CI 32–40) and 71% (95% CI 66–74). Reviewing clinicians diagnosed chronic bronchitis in 25% (95% CI 21–29), and although reviewing clinicians diagnosed COPD in 7.9% (95% CI 5.7–10.6) of patients, spirometry consistent with COPD was present in 35% (95% CI 31–40). A further aim was to determine, using spirometry, whether the substantial levels of restrictive lung function reported in community surveys in sub-Saharan Africa would be evident in everyday clinical practice. Although 38% (95% CI 33–43) of the patients had evidence of restrictive lung function, in nearly 60% there was concomitant fixed airflow obstruction. Purely restrictive spirometry was present in 17% (95% CI 14–21) of patients and not associated with an excess of symptoms.

Depending on strictness of spirometric classification, between 27% and 33% of patients with clinician-diagnosed asthma had spirometry consistent with asthma; however, it is recognised that objective measures of airflow obstruction correlate poorly with asthma symptoms and response to treatment.^25^ We also found that 39% of clinician-diagnosed asthma patients fulfilled the criteria for COPD, and while this may be a consequence of chronic airway remodelling, this finding raises the probability of COPD being misdiagnosed as asthma, a well-recognised issue when diagnoses are totally reliant on symptoms unsupported by spirometry.^26^ In addition to asthma overdiagnosis, there was also evidence for the converse, with 18–21% (depending on strictness of spirometric classification) of symptomatic patients with no asthma diagnosis having spirometry consistent with asthma; numerically, such patients accounted for more than those with spirometry supportive of their clinician-diagnosed asthma. This is consistent with studies reporting underdiagnosis of asthma if the diagnosis is unsupported by spirometry testing.^26^–^28^

Clinician-diagnosed COPD was reported in 8% of patients; however, only 36% had evidence of fixed airflow obstruction. To note, 36% of symptomatic patients with no COPD diagnosis fulfilled the spirometric criteria for COPD, 45% had been diagnosed with asthma by the reviewing clinician and 47% had severe/very severe airflow obstruction, a proportion consistent with the BOLD study findings.^29^ Patients with undiagnosed COPD (*n* = 139) considerably exceeded those with diagnosed COPD (*n* = 41). This finding in symptomatic patient populations adds to the very limited data on the under-diagnosis of COPD in Africa,^30^ and almost certainly reflects the lack of routine access to spirometry.

In Kenya, the most common abnormal pattern of lung function was obstruction, whereas in Ethiopia and Sudan there was a greater prevalence of restriction than in Kenya, with a predominance of a mixed obstruction/restriction pattern. Pure restriction was also more common in Ethiopia and Sudan than Kenya. When compared with Kenya, in addition to having higher rates of diagnosed asthma or COPD, Ethiopia and Sudan also appeared to have higher rates of undiagnosed asthma or COPD. These differences are likely to reflect between-country differences in influential factors accumulated through the life course, e.g., prematurity, birth weight, infant weight gain, diet, air pollution, smoking, occupation(s), TB.

The findings of our study differ from those of a previous study of patients attending clinics in Khartoum, Sudan, that reported 3% of patients had pure obstruction, 6% mixed obstruction/restriction and 5% pure restriction; however, this study was conducted in patients with previous smear-positive TB.^31^ Our findings from Kenya are similar to those of 762 patients referred to the respiratory units of a teaching hospital in Ghana, which had a predominance of obstructive lung disease, with 48% of patients having normal spirometry, 26% pure obstruction, 15% pure restriction and 12% mixed obstruction/restriction.^32^ In a Ugandan study, 17% of 792 patients attending a hospital chest clinic were reported to have diagnosed asthma; however, no spirometry was available.^33^ We found that BD reversibility was more prevalent in Ethiopia (35%) than in Kenya (24%), which is consistent with the findings of the African Severe Asthma Study Project (ASAP) conducted in Ethiopia, Kenya and Uganda.^34^

Our study had a number of strengths. Consecutive patients with respiratory symptoms for more than 8 weeks were recruited, the diagnosis of the reviewing clinician was ascertained, and TB was excluded on clinical grounds and a negative GeneXpert test. We were able to conduct high-quality pre- and post-BD spirometry in the majority of patients, giving us insight into COPD and asthma prevalence. Although we did not achieve our recruitment target of 200 in Ethiopia and Sudan, the sample sizes were based on very conservative 5–10% prevalence rates for asthma and COPD; in reality, the prevalence rates for asthma and COPD were three times higher.

## CONCLUSION

This hospital outpatient clinic-based study in three African countries shows that airway diseases account for the majority of diagnoses in patients with chronic respiratory symptoms (>8 weeks) in whom TB has been excluded. Our findings indicate an unmet clinical need in symptomatic patients to diagnose and manage asthma and COPD in Ethiopia, Kenya and Sudan. Further work is required to identify the clinical CRD burden in other African countries.
